# Hepatitis B Precipitating Neurological Complications: A Chronic Inflammatory Demyelinating Polyneuropathy (CIDP) Conundrum

**DOI:** 10.7759/cureus.53551

**Published:** 2024-02-04

**Authors:** Gaayathri Krishnan, Kiran Kishor Chandrasekar, Ganesh Kumar Natarajan

**Affiliations:** 1 Internal Medicine, AdventHealth Florida, Sebring, USA; 2 Neurology, University Hospital Ayr, Ayr, GBR; 3 Miscellaneous, AdventHealth Florida, Sebring, USA

**Keywords:** neuron, liver, viral hepatitis, hepatitis b, chronic inflammatory demyelinating polyneuroradiculopathy (cidp)

## Abstract

Hepatitis B virus stands as a prominent contributor to cirrhosis, hepatocellular carcinoma, and other liver-related fatalities. On the other hand, neurological manifestations in HBV-infected individuals are infrequently observed. Chronic inflammatory demyelinating polyneuropathy (CIDP) represents an immune-mediated neuropathy, known for its distinctive pattern of symmetrical involvement and weakness in both proximal and distal muscles.

In this study, we present a noteworthy instance of chronic inflammatory demyelinating polyneuropathy (CIDP) occurring in a patient with chronic inactive hepatitis B infection.

## Introduction

Hepatitis B, caused by the hepatitis B virus (HBV), remains a significant global health concern due to its association with cirrhosis, hepatocellular carcinoma, and liver-related mortality. This viral infection affects millions worldwide, posing a substantial burden on healthcare systems and impacting individuals' quality of life [1].

Meanwhile, chronic inflammatory demyelinating polyneuropathy (CIDP) represents a distinct neurological disorder characterized by chronic inflammation of peripheral nerves' myelin sheath. CIDP is an immune-mediated neuropathy known for its progressive and symmetric weakness, sensory abnormalities, and impaired nerve conduction [2].

Hepatitis B infection can lead to extrahepatic manifestations, affecting approximately 1-10% of patients with HBV. These manifestations encompass a range of conditions, such as serum-sickness-like syndrome, polyarteritis nodosa, membranous glomerulonephritis, and papular acrodermatitis of childhood (Gianotti-Crosti syndrome); these are mainly understood to be immune complex-mediated illnesses [1]. Despite its primary impact on the liver and some extrahepatic manifestations, emerging evidence points towards potential neurologic consequences of HBV infection. Neurological manifestations associated with hepatitis B are infrequently observed, but some studies have reported their occurrence in a small subset of patients infected with HBV. Neuropathy is an uncommon occurrence during acute hepatitis B infection but may develop in around 5% of patients with chronic HBV infection [3].

This case report aims to explore the relationship between hepatitis B and CIDP, shedding light on the potential neurological implications of HBV infection. By gaining a deeper understanding of this intriguing interplay between viral hepatitis and immune-mediated neuropathies, we hope to pave the way for improved diagnosis, treatment, and patient outcomes in individuals affected by these complex medical conditions.

## Case presentation

A 68-year-old Hispanic male presented to the hospital with a three-month history of muscle weakness affecting all four extremities. He also endorsed tingling and numbness in his hands and legs. The weakness gradually worsened, involving both proximal and distal muscle groups symmetrically. However, he stated that he had acute onset weakness of the right upper extremity causing him significant distress, which prompted admission and further workup to rule out acute neurological disease.

The patient reported no recent bowel or bladder changes and exhibited no symptoms indicative of respiratory distress. Additionally, he had no complaints of visual symptoms, fevers, chills, or altered mental status. Before hospitalization, the patient could live independently and perform all activities of daily living by himself.

His past medical history was significant for type II diabetes mellitus, poorly controlled on oral hypoglycemic agents, coronary artery disease with recent percutaneous intervention with stenting on dual antiplatelets, and a history of a cerebrovascular accident that left him with right-sided residual deficits. The patient admitted to smoking around half a pack a day for the last 40 years of his life, although he denied alcohol or recreational drug use. He had no significant neurological history in his family.

On examination, he appeared frail. The patient was hemodynamically stable, and cardiovascular, abdominal, and respiratory systems appeared normal. However, his neurological examination was significant for absent deep tendon reflexes in all four limbs, reduced muscle power graded 3/5 in both upper and lower limbs, with proximal muscles showing more significant weakness. He also had a wrist drop, mild muscle wasting in the right hand, and sensory impairment to vibration and position sense. Cranial nerves were intact and there were no signs of dysautonomia on clinical examination.

His admission laboratory values (Table 1) were significant for high random blood sugar, with HbA1c of 8.8 g/dL, and deranged liver function tests with a mixed pattern of injury (AST 40U/L, ALT 78 U/L, alkaline phosphatase 164 U/L, and total bilirubin 1.28 mg/dL).

**Table 1:** Laboratory Values on Admission Lab values showing abnormal liver function tests on admission. AST: aspartate aminotransferase; ALT: alanine transaminase; BUN: blood urea nitrogen; eGFR: estimated glomerular filtration rate

Parameters	Reference ranges	Admission labs
Comprehensive metabolic panel		
AST (U/L)	10-42	40
ALT (U/L)	10-40	78
Alkaline phosphatase (U/L)	55-149	164
Total bilirubin (U/L)	0.00-1.2	1.28
Sodium (mmol/L)	136-145	139
Potassium (mmol/L)	3.5 - 5.0	3.6
Chloride (mmol/L)	98 - 107	102
Bicarbonate (mmol/L)	22.0 - 29.0	26.3
Creatinine (mg/dL)	0.50 - 1.20	0.71
BUN (mg/dL)	6.0 - 20.0	17.8
eGFR (mL/min)	>60	99
Complete blood count		
White blood cells (10*3/uL)	3.70 - 10.30	6.25
Red blood cells (10*6/uL)	4.63 - 6.08	4.68
Hemoglobin (g/dL)	13.7 - 17.5	14.5
Platelets (10*3/uL)	150 - 400	177
Endocrine		
Random glucose (mg/dL)	70-100	227
HbA1c (g/dL)	0.0 - 6.0	8.8
Thyroid-stimulating hormone (u[IU]/mL)	0.340 - 5.600	1.632

A CT scan of the head without contrast was done to rule out any acute neurological issue, which was negative.

In the hospital, the patient underwent an extensive evaluation to identify the cause of his subacute muscle weakness and to rule out possible stroke.

Possible causes investigated included paraneoplastic processes, inflammatory and autoimmune syndromes, HIV-AIDS, syphilis, and metabolic and infectious causes. However, all the tests proved negative, except for the hepatitis panel, which revealed hepatitis B positivity with high viral titers and hepatitis B e antigen (HBeAg) positivity (Table 2).

**Table 2:** Other Laboratory Values Full in-hospital evaluation ANCA: antineutrophil cytoplasmic antibodies; IFA: immunofluorescence assay; PCR: polymerase chain reaction; VDRL: venereal disease research laboratory test; CSF: cerebrospinal fluid

OTHER LABORATORY VALUES	RESULTS
Autoimmune panel	
ANCA IFA pattern	None detected
ANCA IFA titer	<1:20
Anti-DNA antibody, double-stranded	None detected
Anti-Smith antibody	None detected
Striated muscle antibodies, IgG	<1:40
Titin antibody	0.43
Myelin basic protein	2.75 ng/mL
Purkinje cell/neuronal nuclear IgG	None detected
Hepatıtıs panel	
Hepatitis B E antibody	Negative
Hepatitis B E antigen	Positive
Hepatitis B surface antigen	Positive
Hepatitis B core antibody IgM	Negative
HBV DNA, quantitative PCR (<=10 IU/mL)	>100,000,000 IU/m
Hepatitis B PCR, IU/mL	>8.00
Hepatitis A IgM	Nonreactive
Hepatitis C Ab	Negative
Infectious diseases	
HIV1/2 Ag/Ab	Negative
Treponema pallidum (VDRL), serum	Nonreactive
Herpes virus 6 PCR	Not determined
Herpes virus 6 PCR (copy/mL)	<1000 copy/mL
HHV6 PCR (log copy/mL)	<3.0 log copy/m
Meningoencephalitis panel	Negative
Paraproteinemias	
Immunoglobulin G index, CSF	0.64
IgG synthesis rate, CSF	62.8 mg/d
IgG/albumin ratio, CSF	0.61
Kappa/lambda ratio	1.32
Microalb/creat ratio, urine	27.41 mg/g
Microalbumin, urine	2.40 mg/dL

MRI brain showed no evidence of acute ischemic stroke; it was only positive for atrophy and small vessel disease.

CT angiogram brain and neck showed no significant or critical stenosis nor did the carotid ultrasound show any significant or critical stenosis. An echocardiogram of the heart showed a normal ejection fraction of 50-55%, with no evidence of a cardiac source of embolism. An MRI C-Spine showed spinal stenosis, greatest in C6-C7.

Unfortunately, due to the patient's high risk of bleeding while being on dual antiplatelets, a lumbar puncture was not performed in the hospital. Still, it was performed in the outpatient department, which revealed high proteins in the cerebrospinal fluid (CSF) with a normal white cell count, and classical albumin-cytological dissociation (Table 3).

**Table 3:** CSF studies CSF studies with albumins-cytological dissociation. CSF: cerebrospinal fluid; HSV: herpes simplex virus

CSF STUDIES FROM LUMBAR PUNCTURE	RESULTS
Appearance, CSF	Clear
White blood cells, CSF	1 /uL
Red blood cells, CSF	0 /uL
Glucose, CSF	94 mg/dL
Albumin Index	27.5
Albumin, CSF	74 mg/dL
Angiotensin-converting enzyme, CSF	2.0 u/L
HSV 1/2 IgM, CSF	0.27 IU
Immunoglobulin G, CSF	45.2 mg/dL
Total protein, CSF	193.0 mg/dL (High)

In light of the findings, the patient was administered intravenous immunoglobulin (IVIG) for suspected CIDP.

After five days of IVIG treatment, the patient showed complete resolution of his symptoms.

## Discussion

CIDP is an immune-mediated peripheral neuropathy distinguished by progressive motor and sensory impairment stemming from inflammatory demyelination of peripheral nerves [4]. CIDP can have acute onset linked to antecedent infections via molecular mimicry [5-7]. Specifically, several case reports describe acute onset CIDP (A-CIDP) associated with acute HBV infection. The systemic inflammation during acute HBV may trigger aberrant autoimmunity against myelin antigens [8].

Molecular mimicry between HBV and myelin epitopes can lead to autoreactive T cells and antibodies that cross-react with peripheral nerve myelin components like P0, P2, and PMP22. This instigates immune-mediated nerve damage and demyelination, manifesting as A-CIDP [9,10].

Patients with A-CIDP in the setting of acute HBV tend to follow a monophasic disease course rather than the traditional relapsing-remitting or progressive chronic pattern [11,12]. This suggests acute HBV infection induces a distinct CIDP phenotype and clinical presentation.

This patient exhibited features clinically consistent with CIDP. Lumbar puncture and cerebrospinal fluid analysis can support a CIDP diagnosis with classical albumin-cytological dissociation. An elevated CSF protein in patients with diabetes mellitus should be attributed to CIDP if greater than 100 mg/dL, which was revealed in our patient's CSF results. A nerve biopsy, however, could not be obtained to assess nerve histopathology and confirm inflammatory demyelination [13].

Furthermore, the patient had pre-existing diabetes mellitus, which can manifest similar neuropathies [14]. Nerve conduction studies help differentiate CIDP from diabetes-associated neuropathies but were not conducted [15].

Overall, while acute HBV infection may have precipitated an acute onset CIDP-like neuropathy in this case, the diagnosis cannot be definitively established given limitations in performing additional diagnostic testing. Further investigations on the association between acute HBV infection and CIDP with comprehensive diagnostic workup are warranted.

## Conclusions

In conclusion, the interplay between CIDP and acute HBV infection presents a complex narrative that underscores the intricate relationship between immune responses and neuropathological manifestations. The phenomenon of molecular mimicry emerges as a key protagonist, linking the viral realm with the myelin landscape and setting the stage for a symphony of autoimmunity. The fusion of HBV and myelin epitopes catalyzes the emergence of autoreactive agents that, unfortunately, take center stage in the damaging demyelination of peripheral nerves. The patient under scrutiny showcases features that are emblematic of CIDP, yet the definitive diagnosis remains veiled, ensnared by the limitations imposed by diagnostic methodologies. The absence of certain diagnostic procedures, such as MRI of the spine and nerve biopsy, casts shadows of uncertainty on the narrative's resolution, leaving a lingering sense of enigma. The subplot of pre-existing diabetes mellitus introduces further layers of complexity, intertwining the threads of neuropathy and challenging the diagnostic path. The narrative arc highlights the significance of nerve conduction studies in drawing clear distinctions between CIDP and diabetes-associated neuropathies, a facet sadly neglected in this tale.

In conclusion, the call for a comprehensive diagnostic journey becomes resoundingly clear. The nexus between acute HBV infection and CIDP beckons for further exploration through robust diagnostic investigations, casting a spotlight on a potential association that could reshape our understanding of both conditions. In the grand tapestry of medicine, the synthesis of infection and autoimmunity, painted against the canvas of neuropathy, unveils a narrative deserving of continued investigation. The resolution of this enigma awaits the diligent unraveling of diagnostic limitations, inviting future researchers to illuminate the hidden corners of this intricate condition.
